# Hepatitis B Vaccination Impact and the Unmet Need for Antiviral Treatment in Blantyre, Malawi^[Author-notes jiab562-FM1]^

**DOI:** 10.1093/infdis/jiab562

**Published:** 2021-11-09

**Authors:** Alexander J Stockdale, James E Meiring, Isaac T Shawa, Deus Thindwa, Niza M Silungwe, Maurice Mbewe, Rabson Kachala, Benno Kreuels, Pratiksha Patel, Priyanka Patel, Marc Y R Henrion, Naor Bar-Zeev, Todd D Swarthout, Robert S Heyderman, Stephen B Gordon, Anna Maria Geretti, Melita A Gordon

**Affiliations:** Malawi-Liverpool-Wellcome Trust Programme, Blantyre, Malawi; Institute of Infection, Veterinary and Ecological Sciences, University of Liverpool, Liverpool, United Kingdom; Malawi-Liverpool-Wellcome Trust Programme, Blantyre, Malawi; Oxford Vaccine Group, Department of Paediatrics, University of Oxford, Oxford, United Kingdom; Malawi-Liverpool-Wellcome Trust Programme, Blantyre, Malawi; Faculty of Biomedical Science and Health Professions, University of Malawi College of Medicine, Blantyre, Malawi; Malawi-Liverpool-Wellcome Trust Programme, Blantyre, Malawi; Department of Infectious Disease Epidemiology, London School of Hygiene and Tropical Medicine, London, United Kingdom; Malawi-Liverpool-Wellcome Trust Programme, Blantyre, Malawi; Malawi-Liverpool-Wellcome Trust Programme, Blantyre, Malawi; Malawi Ministry of Health, Capitol Hill, Lilongwe, Malawi; Faculty of Medicine, University of Malawi College of Medicine, Blantyre, Malawi; Department of Tropical Medicine, Bernhard Nocht Institute for Tropical Medicine, Hamburg, Germany; 1st Department of Medicine, University Medical Centre Hamburg-Eppendorf, Hamburg, Germany; Malawi-Liverpool-Wellcome Trust Programme, Blantyre, Malawi; Malawi-Liverpool-Wellcome Trust Programme, Blantyre, Malawi; Malawi-Liverpool-Wellcome Trust Programme, Blantyre, Malawi; Liverpool School of Tropical Medicine, Liverpool, United Kingdom; International Vaccine Access Center, Johns Hopkins Bloomberg School of Public Health, Baltimore, Maryland, USA; Malawi-Liverpool-Wellcome Trust Programme, Blantyre, Malawi; National Institute for Health Research Global Health Research Unit on Mucosal Pathogens, Research Department of Infection, Division of Infection and Immunity, University College London, London, United Kingdom; National Institute for Health Research Global Health Research Unit on Mucosal Pathogens, Research Department of Infection, Division of Infection and Immunity, University College London, London, United Kingdom; Malawi-Liverpool-Wellcome Trust Programme, Blantyre, Malawi; Liverpool School of Tropical Medicine, Liverpool, United Kingdom; Department of Infectious Diseases, Fondazione Policlinico Tor Vergata, Faculty of Medicine, University of Rome Tor Vergata, Rome, Italy; Malawi-Liverpool-Wellcome Trust Programme, Blantyre, Malawi; Institute of Infection, Veterinary and Ecological Sciences, University of Liverpool, Liverpool, United Kingdom

**Keywords:** hepatitis B, vaccination, epidemiology, antiviral agents, Malawi, Africa, south of the Sahara, public health

## Abstract

**Background:**

Hepatitis B is the leading cause of cirrhosis and liver cancer in sub-Saharan Africa. To reduce mortality, antiviral treatment programs are needed. We estimated prevalence, vaccine impact, and need for antiviral treatment in Blantyre, Malawi.

**Methods:**

We conducted a household study in 2016–2018. We selected individuals from a census using random sampling and estimated age-sex-standardized hepatitis B surface antigen (HBsAg) seroprevalence. Impact of infant hepatitis B vaccination was estimated by binomial log-linear regression comparing individuals born before and after vaccine implementation. In HBsAg-positive adults, eligibility for antiviral therapy was assessed.

**Results:**

Of 97386 censused individuals, 6073 (median age 18 years; 56.7% female) were sampled. HBsAg seroprevalence was 5.1% (95% confidence interval [CI], 4.3%–6.1%) among adults and 0.3% (95% CI, .1%–.6%) among children born after vaccine introduction. Estimated vaccine impact was 95.8% (95% CI, 70.3%–99.4%). Of HBsAg-positive adults, 26% were HIV-positive. Among HIV-negative individuals, 3%, 6%, and 9% were eligible for hepatitis B treatment by WHO, European, and American hepatology association criteria, respectively.

**Conclusions:**

Infant HBV vaccination has been highly effective in reducing HBsAg prevalence in urban Malawi. Up to 9% of HBsAg-positive HIV-negative adults are eligible, but have an unmet need, for antiviral therapy.

## Teaser

In a large census-based community serosurvey in Blantyre, Malawi adult HBsAg prevalence was 5.1%, and 0.3% among vaccine-eligible children. Estimated vaccine impact was 95.8% (95% CI, 70.3%–99.4%). Up to 9% of HBsAg-positive adults had an unmet need for antiviral treatment.

In sub-Saharan Africa, chronic hepatitis B is the leading cause of liver cirrhosis and hepatocellular carcinoma (HCC) [1]. HCC is the second-highest incident cancer in men and fourth among women in the region, and is usually diagnosed at an advanced stage when curative treatment is no longer possible [2, 3]. In 2016, the World Health Assembly set ambitious targets to reduce the incidence of viral hepatitis by 90% and mortality by 65% by 2030, calling for improved efforts to prevent, diagnose, and treat chronic hepatitis B [4]. Modelling studies project that while hepatitis B prevalence will decline among children due to vaccination programs, hepatitis B-related mortality will increase by 2030 in sub-Saharan Africa without implementation of treatment programs for adults [5]. Data from Western and Asian cohorts show that in chronic hepatitis B, antiviral therapy results in several important outcomes, including regression and reversal of liver fibrosis, reduction in HCC incidence, improved survival, and increased quality of life [6–8]. In the Gambia, a community hepatitis B virus (HBV) screen-and-treat strategy was deemed to be feasible and cost-effective [9].

The infant hepatitis B vaccine was introduced into national immunization schedules across sub-Saharan Africa between 1994 and 2014, and in Malawi in 2002. The World Health Organization (WHO) recommends commencing the first dose at birth but this has not been implemented in most sub-Saharan African countries, including Malawi [10]. In 2019, 3-dose coverage starting at 6 weeks of age, was median 87% (interquartile range [IQR], 74–93) across the region and 95% in Malawi [11]. A community assessment of vaccine impact has not previously been conducted in southern Africa, outside of South Africa, and there are therefore no data for Malawi.

In a previous systematic review, we estimated that hepatitis B surface antigen (HBsAg) prevalence was 8% among adults in Malawi, but observed that available data were predominantly based on convenience sampling, hence at significant risk of bias [12]. Estimates of the community-level burden of HBV infection and disease, and the projected need for hepatitis B treatment, are required to inform an effective public health response [4]. To address a significant gap in knowledge, we conducted a census-based serological and liver-disease survey in Blantyre, Malawi to ascertain HBsAg seroprevalence and impact of hepatitis B vaccination, and to estimate the population-level eligibility and unmet need for hepatitis B treatment.

## METHODS

### Census and Serological Survey

Individuals of all ages resident in Ndirande, Blantyre, Malawi were randomly selected from a population demographic census and invited to participate in a serosurvey in 2016–2018. Ndirande is an unplanned urban township in the north of Blantyre. Malawi is a low-income country in southern Africa with a life expectancy at birth of 64 years and national human immunodeficiency virus (HIV) prevalence of 10.6% (95% confidence interval [CI], 9.9%–11.2%) among adults [13].

We used single-stage random probability sampling, with age stratification to oversample younger children, as part of a coincident typhoid epidemiology study (Strategic Typhoid Alliance across Africa and Asia) [14, 15]. Global positioning satellite coordinates of households were recorded (eTrex 30x; Garmin) to assess spatial distribution of selected individuals. If a randomly selected individual in the serosurvey could not be located or did not consent, another household member from the same age-stratification group was requested to participate or, secondarily, a replacement was selected by further randomization from the census age stratum. Educational, marital, and employment data were recorded from serosurvey participants. We estimated a sample size requirement of 5913 based on an anticipated HBsAg prevalence of 8.1% for precision of 1% (Supplementary Material 1). Vaccination status of children aged ≤10 years was obtained from the family-held vaccine record or, if unavailable, parent or guardians’ report. Venous EDTA samples were collected in consenting participants’ households, stored in cool boxes, and transported to the study laboratory. Plasma samples were separated by centrifugation and stored at −80°C.

### Evaluation of HBV Disease

We returned to households of individuals aged ≥16 years who tested HBsAg positive by laboratory enzyme immunoassay and invited them to participate in an evaluation of treatment eligibility based on WHO 2015, European Association for the Study of the Liver 2017 (EASL), and American Association for the Study of the Liver (AASLD) 2018 criteria (Supplementary Material 2) [8, 16, 17]. For pregnant women, for whom transient elastography is not recommended, we returned >6 weeks after delivery to invite them to participate. Inclusion criteria for evaluation of treatment eligibility were HBsAg seropositivity, residence in the study catchment area, and capacity to consent. We conducted assessments in community halls close to participants’ residences. Rapid point-of-care HIV testing was offered in accordance with national guidelines using Determine anti-HIV (Alere) for screening, followed by confirmation with Uni-Gold HIV (Trinity Biotech). We performed clinical examination to elicit signs of chronic liver disease. We assessed liver stiffness using transient elastography in the right midaxillary intercostal space after fasting for >3 hours (FibroScan 430 Mini; Echosens). Reliability criteria were IQR/median <0.3 if >7.1 kPa [18]. We applied categorical interpretative cutoffs of 7.9 kPa for significant fibrosis (F2) and 9.5 kPa for cirrhosis (F4) according to cross-sectional comparative data from the Gambia and Senegal [19]. Participants meeting EASL criteria were referred for treatment with tenofovir.

### Laboratory Investigations

Laboratory investigations were performed in the Malawi-Liverpool-Wellcome Trust Laboratories in Blantyre. We tested for HBsAg using the Monolisa HBsAg-Ultra (Bio-Rad) enzyme-linked immunoassay (ELISA) in accordance with manufacturer’s instructions. Samples showing intermediate reactivity (sample/cutoff ratio >0.9) and all positive samples were repeated in duplicate. Hepatitis B e antigen (HBeAg), anti-HBe, hepatitis C antigen/antibody (HCV Ag/Ab), and anti-hepatitis delta virus (anti-HDV) were tested by ELISA using Monolisa HBeAg-Ab (Bio-Rad), Monolisa HCV Ag-Ab ULTRA v2, (Bio-Rad), and ETI-AB-DELTAK-2 (Diasorin), respectively. HBV DNA was quantified using an in-house real-time polymerase chain reaction (PCR), with a lower limit of quantification of 34 IU/mL (Supplementary Material 3). Alanine transaminase (ALT) was measured using an automated assay with an upper limit of normal of 32 U/L (AU480; Beckmann Coulter).

### Ethical Review

Ethical permission to conduct the study was obtained from the National Health Sciences Research Committee of Malawi (16/11/1698 and 15/5/1599) and the University of Liverpool (reference 1954). Participants in the serological survey and clinical evaluation provided written informed consent. The study was conducted in accordance with the Declaration of Helsinki, 2013.

### Statistical Analysis

We estimated population HBsAg prevalence using survey design weights from age stratification and used poststratification iterative proportional fitting with 5-year age-sex groups from the census to adjust estimates to the population distribution. In a sensitivity analysis, we also adjusted for geographic area to account for geographic variation in response rate. Where date of birth was unknown, we estimated the year midpoint from reported age. We assessed spatial clustering of HBsAg-positive participants using Getis-Ord Gi∗ and Anselin Local Moran I statistics. Association between HBsAg and explanatory variables were assessed by binomial logistic regression, applying sampling probability weights. For multivariable model selection among participants aged ≥16 years we considered variables with *P*<.25 in univariable analysis for inclusion and included a restricted cubic spline for age to account for variation in HBsAg prevalence with respect to age. To calculate socioeconomic status (SES), we analyzed responses to a household economic survey from 12080 households (Supplementary Material 4) using principal component analysis to derive relative wealth quintiles for 44 areas represented in the serosurvey [20]. To estimate vaccine impact, we compared individuals born within 5 years prior to vaccine introduction in 2002 (aged between 15 and 21 years at the date of sampling) with those born within 5 years after vaccine introduction (aged 10 to 16 years), and in a sensitivity analysis, compared those born within 10 years before and after implementation. Ages in the 2 groups overlapped due to the 18-month duration of the serosurvey. Vaccine impact was calculated as (1 − risk ratio) × 100, where the risk ratio was estimated from binomial log-linear regression, adjusted for survey weights. In sensitivity analyses, we compared the primary model with Poisson regression with robust standard errors and logistic regression models, and assessed the effect of adding participant age at sampling and 2-year birth cohort intervals as covariates using the Wald test, to assess for an age-cohort effect. Analyses were conducted in ArcGIS Pro version 2.4.1 (Esri) and Stata version 16.1 (Statacorp).

## RESULTS

### HBsAg Seroprevalence and Vaccine Impact

**Figure 1.**
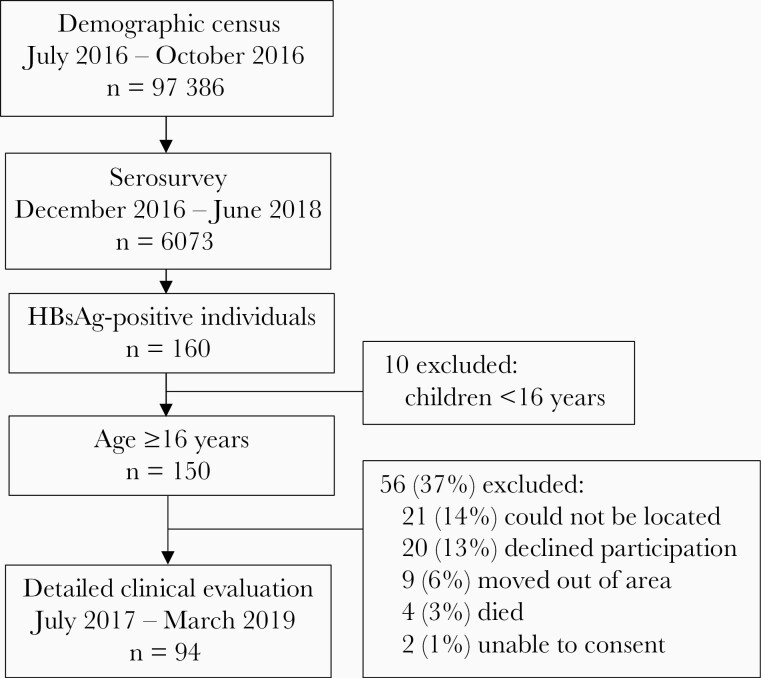
Flowchart of census and recruitment to the serological survey and community hepatitis B virus treatment evaluation study. Abbreviation: HBsAg, hepatitis B surface antigen.

**Figure 2.**
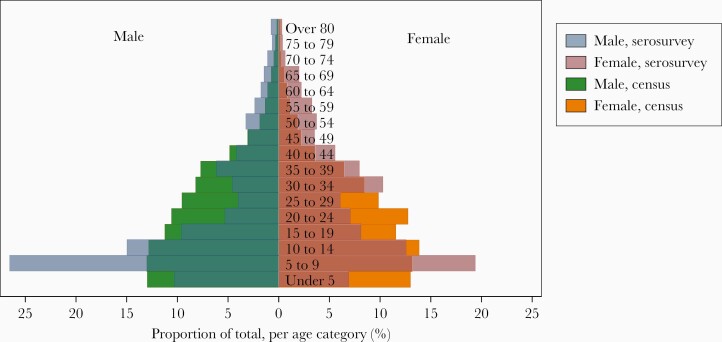
Distribution of age (in years) and sex in the serosurvey relative to the demographic census. Serosurvey age distribution is layered over census distribution data.

**Figure 3.**
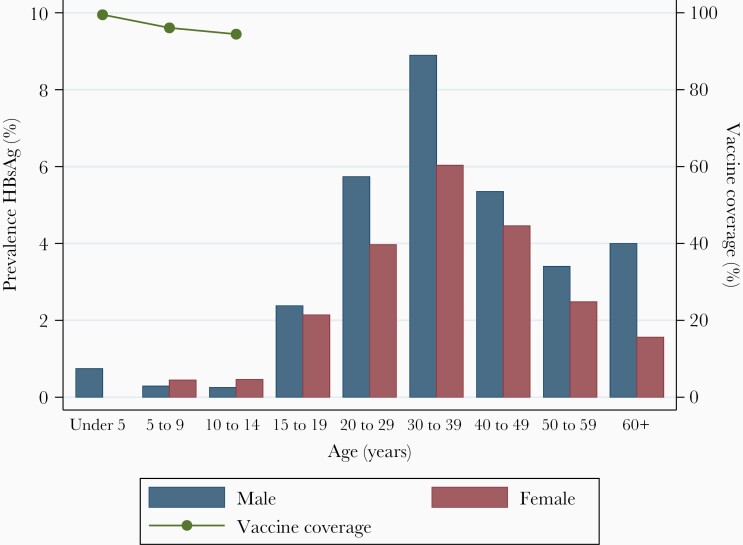
Prevalence of hepatitis B surface antigen stratified by age and sex groups and hepatitis B virus vaccine coverage. Vaccine coverage is shown among serosurvey participants born after implementation of the vaccine in 2002. Tabulated age and sex stratified prevalence data are shown in Supplementary Material 6. Abbreviation: HBsAg, hepatitis B surface antigen.

Overall, 6073 individuals participated in the serological survey and were tested for HBsAg, from a total census population of 97386 (Figure 1). Median age of serosurvey participants was 18 years (IQR, 8–37) and 3455/6073 (56.7%) were female. This compares to a median age of 17 years (IQR, 4–32) and a female proportion of 51.5% in the national 2018 census [21]. The serosurvey oversampled younger children and older adults relative to the census distribution (Figure 2). A total of 160/6073 (2.6%) participants tested positive for HBsAg. No spatial clustering of HBsAg-positive individuals was observed with Getis-Ord Gi∗ and Anselin Local Moran I statistics (Supplementary Material 5). The age- and sex-standardized prevalence of HBsAg in the general population was 3.1% (95% CI, 2.6%–3.7%). Prevalence peaked among males aged 30–39 years and declined among older adults (Figure 3 and Supplementary Material 6).

Vaccination status was obtainable from 1172/2085 (56.2%) children aged ≤10 years and 631/931 (67.8%) children aged ≤5 years. Data sources comprised health records for 722/1172 (61.6%) and parent/guardian report for 450/1172 (38.4%). Completion of 3-dose HBV vaccination was reported for 1141/1172 (97.4%) children aged ≤10 years and 619/631 (98.1%) among children aged ≤5 years, for whom vaccine status was known. Vaccine coverage was associated with data source, with 96.5% reporting complete vaccination from health records and 98.6% from oral parental/guardian report (*P*=.03). Only 31 children were ascertained to be unvaccinated and none tested HBsAg positive. Standardized HBsAg prevalence was 0.6% (95% CI, .2%–1.4%) among 913 children with unknown vaccination status, which was not significantly different from children completing all 3 doses (0.2%; 95% CI, .1%–.8%; *P*=.26).

Among children born after infant vaccine implementation in 2002, standardized HBsAg prevalence was 0.3% (95% CI, .2%–.6%), while among all adolescents and adults born prior to vaccine introduction it was 5.1% (95% CI, 4.3%–6.1%; Table 1). Vaccine impact was 95.9% (95% CI, 70.6%–99.4%) in the primary analysis, comparing individuals born in the 5 years prior to vaccine introduction (aged 15–21 years) with those born in the 5 years afterwards (aged 10–16 years). In a sensitivity analysis, comparing those born 10 years before and after vaccine introduction, vaccine impact was 93.3% (95% CI, 83.1%–97.4%). Sensitivity analyses including Poisson regression with robust standard errors and logistic regression models were consistent with the primary model (Supplementary Material 7). Inclusion of age and cohort variables did not indicate a significant age-cohort effect. Poststratification adjustment for geographic area did not affect prevalence estimates (Supplementary Material 8). By univariate analysis, HBsAg-positive individuals aged ≥16 years were more likely to be male, in paid or self-employment, and to be married, separated, or divorced. No association with educational attainment or regional SES status was observed. In a multivariable model, greater odds of HBV infection were observed in males and separated or divorced individuals (Table 2).

**Table 1.** Prevalence of Hepatitis B Surface Antigen Stratified by Birth Date and Vaccination Status

Population	Crude HBsAg Prevalence		Population Standardized HBsAg Prevalence, % (95% CI)^a^	Risk Ratio (95% CI)	*P* Value
	Frequency	% (95% CI)			
All individuals	160/6073	2.6 (2.3–3.1)	3.2 (2.7–3.7)		
Birth date relative to vaccine introduction^b^					
5 y before vaccine	15/500	3.0 (1.8–4.9)	2.9 (1.8–4.7)	Reference	
5 y after vaccine	1/758	0.1 (.02–.7)	0.1 (.02–.8)	0.04 (.06–.29)	.001
10 y before vaccine	30/884	3.4 (2.4–4.8)	3.6 (2.5–5.1)	Reference	
10 y after vaccine	5/1932	0.3 (.1–.6)	0.2 (.1–.6)	0.07 (.03–.17)	<.0001
All before vaccine	152/3280	4.6 (4.0–5.4)	5.1 (4.3–6.0)	Reference	
All after vaccine	9/2793	0.3 (.2–.6)	0.3 (.2–.6)	0.06 (.03–.12)	<.0001
Vaccination status for age ≤10 y					
Completed 3 doses	3/1141	0.3 (.1–.8)	0.2 (.1–.8)	Reference	
Unknown status^c^	5/913	0.5 (.2–1.3)	0.6 (.2–1.4)	2.3 (.5–10.2)	.26
Incomplete^d^	0/31	0.0 (.0–11.0)	…	…	

Abbreviations: CI, confidence interval; HBsAg, hepatitis B surface antigen.

Standardized to census age and sex distribution.

Hepatitis B vaccination was introduced on 1 January 2002.

Participants born after vaccine introduction for whom vaccination status could not be ascertained from parent, guardian, or documentation; vaccine status was ascertained for 1172/2085 (56.2%) children aged ≤10 years.

Received 0, 1, or 2 doses.

**Table 2.** Participant Characteristics Associated With Hepatitis B Infection in the Serosurvey: Binomial Logistic Regression Model

Characteristic	Univariate Odds Ratio (95% CI)	*P* Value	Multivariable Model, Odds Ratio (95% CI)^a^	*P* Value
Age, per y	1.03 (1.03–1.04)	<.001	^a^	
Age group, y		<.001		<.001
0–14	0.09 (.04–.18)		…	
15–29	Reference		Reference	
30–44	1.82 (1.22–2.73)		1.80 (.87–3.72)	
45–59	0.81 (.45–1.46)		1.49 (.56–3.98)	
>60	0.74 (.35–1.56)		0.55 (.20–1.50)	
Birth date		<.001		
Born prior to vaccine	Reference			
Born after vaccine	0.06 (.03–.11)			
Sex, male vs female	1.46 (1.04–2.03)	.03	1.60 (1.06–2.41)	.02
Marital status^b^		.08		.21
Single	Reference		Reference	
Married	1.58 (1.00–2.49)		1.51 (.75–3.02)	
Separated/divorced	2.73 (1.12–6.64)		2.89 (.96–8.67)	
Widowed	0.96 (.39–2.36)		1.67 (.53–5.22)	
Education^b^		.79		
Primary	Reference			
Secondary	1.07 (.72–1.59)			
Vocational	0.63 (.23–1.78)			
University	1.44 (.57–3.61)			
None	1.36 (.37–4.96)			
Employment^b^		.01		
Unemployed	Reference			
Student	0.93 (.40–2.16)			
Self-employed	2.44 (1.28–4.64)			
Paid employee	2.20 (1.11–4.38)			
Unpaid family worker	1.30 (.62–2.72)			
Retired	1.65 (.22–12.60)			
Socioeconomic status^c^		.49		
Highest quintile	Reference			
2nd highest quintile	1.21 (.62–2.37)			
Middle quintile	0.73 (.36–1.49)			
2nd poorest quintile	1.11 (.57–2.17)			
Poorest quintile	1.10 (.56–2.17)			

Multivariable model considers individuals aged ≥16 years and includes a cubic spline variable for age to account for change in prevalence with respect to age.

Education, employment, marital status, and socioeconomic status data are applicable to individuals aged >16 years.

Socioeconomic quintiles were derived for the 44 health surveillance areas in the serosurvey in which the participants resided.

### Treatment Eligibility

**Figure 4.**
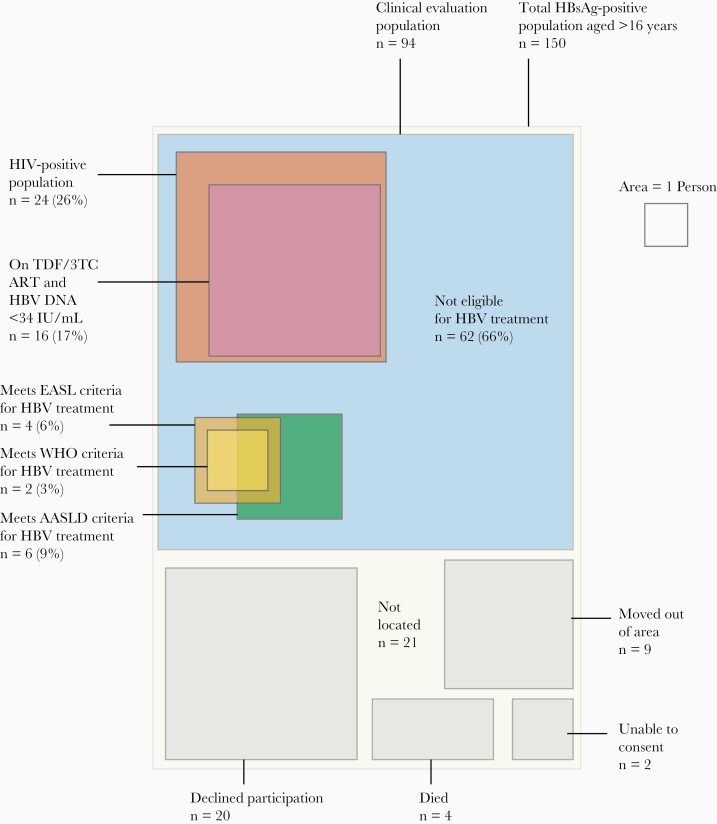
Outcomes of community clinical evaluation for HBV treatment eligibility. Area is proportional to the number of people in each group. Treatment eligibility criteria are considered only for HIV-negative individuals, as all HIV-positive people should receive ART containing TDF/3TC. Abbreviations: 3TC, lamivudine; AASLD, American Association for the Study of the Liver; ART, antiretroviral therapy; EASL, European Association for Study of the Liver; HBsAg, hepatitis B surface antigen; HBV, hepatitis B virus; HIV, human immunodeficiency virus; TDF, tenofovir disoproxil fumarate; WHO, World Health Organization.

Evaluation for treatment eligibility occurred a median of 4.9 months (IQR, 2.2–7.6 months) after the serosurvey. Of 150 HBsAg-positive serosurvey participants aged ≥16 years, we were able to locate 114 in the township (76.0%); 4 had died, 2 were unable to provide consent, and 9 had moved outside the region (Figure 4). A total of 94/114 (82.5%) eligible individuals agreed to participate in evaluation of treatment eligibility. Participants had a higher level of education, higher SES, and higher rate of employment relative to nonparticipants (Supplementary Material 9).

Characteristics of the clinical evaluation population are shown in Table 3. Of 93/94 (98.9%) who agreed to HIV testing, 24/93 (25.8%) were HIV positive, of which 7 (29.1%) were newly diagnosed. Median CD4 count was 519 cells/mm^3^ (IQR, 412–577). Of those with HIV/HBV coinfection, 16/24 (67%) were on antiretroviral therapy (ART), all of which contained HBV-active agents including tenofovir, and all 16 had HBV DNA suppression <34 IU/mL. Anti-HDV was positive in 2/94 (2%) and HCV Ag/Ab was negative in all participants. HBeAg was positive in 10/94 (11%): 25% (6/24) among HIV-positive participants and 6% (4/69) among HIV-negative participants (*P*=.02). In the HIV-negative population, HBV DNA was >2000 IU/mL in 28% (19/69) and >20000 IU/mL in 16% (11/69), and 3% (2/69) had an ALT above the upper limit of normal. Liver stiffness exceeding 9.5 kPa was observed in 3/69 (4%) of the HIV-negative and 1/24 (4%) of the HIV-positive population. Following clinical evaluation of the HBsAg-positive and HIV-negative population using WHO, EASL, and AASLD criteria, 2.9%, 5.7%, and 8.7% met eligibility criteria for HBV treatment, respectively (Figure 4). Projecting EASL criteria to the national population of 17.6 million, this represents an estimated 25586 individuals (95% CI, 7172–65519) requiring HBV treatment (Supplementary Material 10).

**Table 3.** Characteristics of Participants and Outcome of Community Evaluation of HBV Treatment Eligibility

Characteristic	All Participants (n=94)	HIV-Positive Population^a^ (n=24)	HIV-Negative Population (n=69)
Age, y, median (IQR)	36 (29–41)	39 (36–47)	34 (27–38)
Sex, female	49 (52)	14 (58)	35 (51)
CD4 count, cells/mm^3^, median (IQR)^b^		519 (412–577)	
On ART		16/24 (67)	
On TDF or 3TC		16/24 (67)	
Signs of CLD	3 (3)	1 (4)	2 (3)
Past medical history			
History of tuberculosis	7 (7)	6 (25)	1 (1)
Diabetes	1 (1)	0 (0)	1 (1)
Renal disease	1 (1)	0 (0)	1 (1)
Hypertension	1 (1)	0 (0)	1 (1)
Alcohol consumption			
Abstinent	59 (63)	12 (50)	47 (68)
Low risk	23 (25)	9 (38)	14 (20)
Hazardous	10 (11)	3 (13)	6 (9)
Harmful	1 (1)	0 (0)	1 (1)
Alcohol dependence	1 (1)	0 (0)	1 (1)
Hepatitis D and C serology			
Anti-HDV	2 (2)	0 (0)	2 (3)
HCV Ag/Ab	0 (0)	0 (0)	0 (0)
Hepatitis B viral markers			
HBeAg	10 (11)	6 (25)	4 (6)
HBV DNA, IU/mL, median (IQR)	81 (<32–801)	<32 (<32–<32)	215 (51–2880)
HBV DNA>2000 IU/mL	19 (20)	0 (0)	19 (28)
HBV DNA>20000 IU/mL	11 (12)	0 (0)	11 (16)
ALT, IU/L, median (IQR)^c^	10 (8–13)	12 (10–15)	9 (8–12)
ALT>ULN	2 (2)	0 (0)	2 (3)
ALT>2× ULN	0 (0)	0 (0)	0 (0)
Liver stiffness, kPa, median (IQR)	4.8 (4.0–6.4)	4.5 (4.0–5.4)	5.1 (4.0–6.9)
≥7.9 kPa	10 (11)	1 (4)	9 (9)
≥9.5 kPa	4 (4)	1 (4)	3 (4)

Data are No. (%) except where indicated.

Abbreviations: 3TC, lamivudine; Ag/Ab, antigen/antibody; ALT, alanine transaminase; ART, antiretroviral therapy; CD4, cluster of differentiation 4; CLD, chronic liver disease; HBeAg, hepatitis B e antigen; HBV, hepatitis B virus; HCV, hepatitis C virus; HDV, hepatitis D virus; HIV, human immunodeficiency virus; IQR, interquartile range; TDF, tenofovir disoproxil fumarate; ULN, upper limit of normal.

One participant did not consent to HIV testing.

CD4 count available for 21/23 (91%) of HIV-positive individuals.

The assay upper limit of normal was 32 U/L.

## DISCUSSION

Fifteen years after implementation of the hepatitis B vaccination program in Malawi, we observed a vaccine impact of 96%, by comparison of people born 5 years before and after vaccine introduction. Coverage exceeding 97% was observed among children for whom vaccination status could be ascertained. These encouraging data show that in the setting of very high vaccine coverage, HBV vaccination has been highly effective, with a low residual HBsAg prevalence (<0.5%) among children born after HBV vaccination was added to the enhanced program of immunization.

There are limited existing data evaluating community HBV vaccine performance from sub-Saharan Africa. In the Gambia, among 753/2670 young adults who could be linked to vaccination data, estimated vaccine efficacy was 94% (95% CI, 77%–99%) [22]. In Senegal, estimated vaccine efficacy was 95% (95% CI, 77%–99%) among 9 to 12-year-old participants of an HBV vaccination study [23]. In a household survey in South Africa, HBsAg prevalence was 0.9% among 15–19 year olds born after vaccine implementation, relative to 2.8% among those born before implementation [24]. In a sample of 18-month-old children attending vaccine clinics in South Africa, HBsAg prevalence was 0.4%, relative to historical prevaccination prevalence of 9.9% in the Eastern Cape [25]. In serosurveys in Ethiopia and Nigeria, in the setting of lower vaccine coverage rates ranging from 55% to 85%, direct vaccine effectiveness estimates ranged from 66% to 81% [26, 27]. Collectively, these studies show vaccination in sub-Saharan Africa has had a significant impact in reducing HBV prevalence among children but also highlight the paucity of adequately powered representative community assessments across much of the region, and the importance of achieving high vaccine coverage. Maternally acquired HBV is associated with an increased risk of chronicity and development of cirrhosis [28], and is not prevented by vaccination beginning at 6 weeks. Evaluation of transmission rates from HBsAg-positive mothers are required to better assess the need for birth-dose vaccination and antenatal maternal antiviral therapy [29].

To date, there are very limited representative data on the unmet need for antiviral therapy at the community level. Based on Malawi’s 2018 census population of 17.6 million people, we estimate that 25586 HIV-negative people (95% CI, 7225–65657) are eligible, and have an unmet need, for HBV treatment by EASL criteria. This compares to an estimated 970000 adults living with HIV of whom 810000 are currently receiving ART [13], highlighting that HBV antiviral treatment is an achievable public health goal. A quarter of HBsAg-positive adults at the community level had HIV coinfection in this study, of whom two-thirds were already receiving HBV-active ART, all with HBV DNA suppression. This is consistent with results of a national HIV survey in which 68% of individuals had HIV virological suppression, demonstrating the remarkable progress Malawi is making toward HIV control [13]. HIV treatment programs represent a model for HBV due to similarities, including the need for lifelong antiviral therapy including tenofovir, and the potential to share clinic staff, facilities, and laboratory equipment [1].

In our population serosurvey, we observed low HBsAg prevalence in vaccine-eligible children, a peak in the 30 to 40-year age group and declining prevalence among older adults. Declining prevalence after 40 years may be due to spontaneous HBsAg clearance, or death from cirrhosis or HCC, or from infections that share epidemiological risk factors [30]. Increasing prevalence between adolescence and 40 years may be the result of incident HBV transmission, or due to HIV prevention messaging such as promotion of condom use among younger cohorts [13]. A detailed understanding of transmission is limited by the cross-sectional design and lack of previous historical data in children for Malawi. A longitudinal cohort or a repeated cross-sectional survey is required to better elucidate trends in transmission. The lack of association we observed between HBV and SES is in contrast to most infectious diseases, where poverty has a causal role. The population under study has high poverty levels and limited intrasample variation in SES could cause a lack of observable association in this sample. Male gender was an independent risk factor for HBV infection among adults, in keeping with other studies from sub-Saharan Africa [31]. Causal mechanisms may include unsafe circumcision, shared razor blades, and use of shared equipment at barbershops [32].

Modelling studies project that HBV antiviral treatment programs are necessary to reduce HBV-associated mortality or mortality will rise beyond 2030, even in the context of effective infant vaccination [5]. Thus, the success of the vaccination program we observed does not abrogate responsibility to tackle the anticipated rise in adult liver disease. In this community, by use of WHO, EASL, or AASLD criteria, 3%, 6%, and 9% HBsAg-positive individuals, respectively, were eligible for treatment [8, 16, 17]. Two previous community studies have assessed HBV treatment eligibility in sub-Saharan Africa, in the Gambia and Zambia. In the Gambia, 4.4% of community participants and 9.7% of HBsAg-positive blood donors were eligible for treatment, based on EASL 2012 criteria [33]. In Zambia, 10% were treatment eligible by WHO criteria and 17% by EASL 2017 criteria [34]. In a hospital-based sample in Ethiopia, 20% had cirrhosis and 25.2% required treatment by EASL 2012 criteria [35]. These compare to estimates from a recent systematic review where, globally, 12% of participants in community studies required treatment [36]. International treatment criteria are derived from European and Asian data, and prospective cohort data from sub-Saharan are required to validate treatment criteria in sub-Saharan Africa, considering that the African region has among the highest prevalence of hepatitis B globally [37]. Recent evidence from Ethiopia observed that WHO guidelines failed to identify as eligible for treatment 36% of patients with compensated cirrhosis, who are likely to benefit most from antiviral treatment [38].

Our study benefitted from significant strengths including a large sample size, an unbiased census-based random population selection method, and the capacity for adjustment for undersampling of harder-to-reach demographic groups using the population census. We also used rigorous methods to assess treatment eligibility in line with international guidelines. There are several limitations to this analysis. First, due to high vaccine coverage we were unable to estimate vaccine effectiveness, with only 31 unvaccinated children identified in the sample. Instead, we estimated vaccine impact, comparing birth cohorts relative to the timing of national vaccine introduction. This approach relies upon an assumption that exposure risk is equivalent between the 2 groups. Prevaccination data among children were not available to facilitate a comparison of age-matched groups [12], although some age overlap between pre- and postvaccination groups did occur due to the 18-month duration of the serosurvey. The study also suffered from a low rate of confirmed vaccine coverage due to limited availability of parent-held vaccination records. Second, our findings may not be nationally or regionally generalizable because we sampled from an urban population where vaccine uptake exceeds national coverage [11]. In the 2018 population census, 84% of Malawians resided in rural areas. Third, reasons for refusal were not recorded during the serosurvey to assess nonresponse bias. We mitigated this potential bias using poststratification weighting to adjust estimates to the population age and sex distribution and did not detect an influence of geographic nonresponse on estimates. Fourth, we were unable to locate a third of HBsAg-positive individuals, limiting the statistical power available to estimate eligibility. Nonparticipants had lower SES and educational attainment, which could have resulted in selection bias and underestimation of liver disease burden. Our cross-sectional assessment may have resulted in further underestimation of the total need for treatment given that parameters used to assess eligibility, such as HBV DNA and ALT, may fluctuate and evolve over time.

In conclusion, in urban Malawi, 15 years of infant HBV vaccination has been associated with a vaccine impact of 96%. Among HIV-negative individuals, antiviral therapy was required in 3%–9% of HBsAg-positive adults, a significant unmet need. This is an achievable public health goal and an opportunity to prevent cirrhosis and reverse trends in rising hepatitis B-associated mortality. Research to evaluate longitudinal performance of treatment eligibility criteria and to support treatment program implementation is required to eliminate HBV as a public health threat in sub-Saharan Africa.

## Supplementary Data

Supplementary materials are available at *The Journal of Infectious Diseases* online. Supplementary materials consist of data provided by the author that are published to benefit the reader. The posted materials are not copyedited. The contents of all supplementary data are the sole responsibility of the authors. Questions or messages regarding errors should be addressed to the author.

jiab562_suppl_Supplementary_Material

## Data Availability

For the purpose of open access, the author has applied a CC BY public copyright license to any Author Accepted Manuscript version arising from this submission.

## Notes

***Acknowledgments***. The authors thank Ndirande community leaders, health surveillance assistants, and the community of Ndirande, Blantyre. We thank the members of the Strategic Typhoid Alliance across Africa and Asia (STRATAA) consortium.

***Disclaimer***. The funders did not have any role in the conduct of the study, writing of the manuscript, or decision to submit for publication.

***Financial support.*** This work was supported by the Wellcome Trust (grant numbers 109130/Z/15/Z Clinical PhD Fellowship to A. J. S. and 106158/Z/14/Z Strategic Award to the STRATAA study); and the Bill and Melinda Gates Foundation (grant number 617 OPP1141321 to the STRATAA study). The Malawi-Liverpool-Wellcome Trust Clinical Research Programme is supported by a Wellcome Trust Major Overseas Programme Core Award.

Presented in part: International Liver Congress, European Association for the Study of the Liver, 27–29 August 2020 (virtual); and the 11th European Congress on Tropical Medicine and International Health, Liverpool, United Kingdom, 16–20 September 2019.
