# Supplementary material for: Hepatitis B Vaccination Impact and the Unmet Need for Antiviral Treatment in Blantyre, Malawi
Source: J Infect Dis. 2021 Nov 9;226(5):871–80. doi: 10.1093/infdis/jiab562 (PMC9470106; doi:10.1093/infdis/jiab562)
Supplement: jiab562_suppl_Supplementary_Material [file jiab562_suppl_supplementary_material.docx]

**Supplementary Appendix 1: Sample size calculation for serosurvey and clinical evaluation population**

| **Population** | **Statistic** | **Reference** |
| --- | --- | --- |
| Estimated adult HBsAg prevalence in Malawi | 8.1% | [12] |
| Estimated prevalence of treatment eligibility among community HBsAg positive participants | 4.4% | [33] |
| Census population size (for finite population) | 97,386 | Study census |

Using the formula:

N= z^2^ p (1-p)
 e^2^

Where the finite population correction is N(adjusted)= N.n
 N+n

Where z is the z-score for the normal distribution corresponding to the desired confidence level, p is the expected true proportion, e represents desired precision, n is the finite population size (the census population) and N is the estimated sample size requirement.

For sample size calculation based on assessment of treatment eligibility, we used prior estimates of adult HBsAg prevalence of 8.1% from a systematic review[12] and an estimated community prevalence of treatment-eligible adults of 4.4% from a community study in the Gambia[33], which we conservatively rounded to 5%. To achieve a desired a precision (half-width) of 3% with 95% confidence intervals, the estimated sample size requirement was 203 HBsAg positive adults. Based on anticipated HBsAg prevalence of 8.1% this would require sampling 2506 adults. The age stratification design of the co-incident STRATAA study serosurvey targeted a sample of 4600 (54.1%) children aged 0-14 years and 3900 (45.9%) ≥15 year old participants.[14] The total estimated sample size requirement for treatment eligibility assessment, adjusting for the age stratification design, was 5462.

For estimation of HBsAg prevalence among participants aged ≥15 years with a precision half width of 1% based on a predicated HBsAg prevalence of 8.1% and with a finite population size adjustment for the census population of 97,386, the estimated sample size requirement of participants aged ≥15 years was 2713. After adjustment for the age stratification design, the total estimated sample size requirement was 5913.

**Supplementary Appendix 2: Comparison of World Health Organization (WHO) 2015, European Association for the Study of the Liver (EASL) 2017 and American Association for the Study of the Liver (AASLD) 2018 criteria for HBV treatment eligibility**

| **Criteria** | **WHO 2015[16]** | **EASL 2017[8]^a^** | **AASLD 2018[15]^a^** |
| --- | --- | --- | --- |
| Cirrhosis | Cirrhosis based on clinical criteria^b^ or APRI score >2.0 | Cirrhosis^c^ | Cirrhosis^c^ |
| HBeAg, ALT and HBV DNA criteria | Age >30 years, persistently abnormal ALT and HBV DNA >20,000 IU/ml^d^ | HBV DNA >2000 IU/ml, ALT>ULN, and moderate liver necroinflammation or fibrosis (≥F2) by NIT/LB | HBeAg positive, ALT ≥2x ULN, HBV DNA > 20,000 IU/ml |
|  |  | HBV DNA >20,000 IU/ml and ALT>2x ULN (regardless of degree of fibrosis) | HBeAg positive, ALT>ULN, HBV DNA >20,000 IU/ml and NIT/LB indicates ≥F2 |
|  |  | Age > 30 years, HBeAg positive with persistently normal ALT + HBV DNA >2000 IU/ml | HBeAg positive, ALT normal, age >40 years, HBV DNA > 1,000,000 IU/ml and NIT/LB indicates ≥F2 |
|  |  | HBV DNA >2000 IU/ml and moderate fibrosis (≥F2) by NIT/LB, regardless of ALT | HBeAg negative, ALT≥2x ULN, HBV DNA > 2,000 IU/ml |
|  |  |  | HBeAg negative, ALT persistently >ULN, HBV DNA >2000 IU/ml and NIT/LB indicates ≥F2 |
| Family history |  | Family history of HCC or cirrhosis |  |

Abbreviations: ALT alanine aminotransferase; HBeAg hepatitis B e antigen; HBV hepatitis B virus; ULN upper limit of normal; NIT non-invasive test eg. transient elastography; LB liver biopsy; HCC hepatocellular carcinoma; APRI aspartate aminotransferase to platelet ratio index

^a^ ALT >35 for males and 24U/L for females is used as the upper limit of normal in AASLD guidelines. For EASL guidelines, the upper limit of normal is 40 U/L. ^b^Clinical criteria comprise clinical features of decompensated cirrhosis: Portal hypertension (ascites, variceal haemorrhage and hepatic encephalopathy), coagulopathy, or liver insufficiency (jaundice). Other clinical features of advanced liver disease/cirrhosis may include: hepatomegaly, splenomegaly, pruritus, fatigue, arthralgia, palmar erythema, and oedema. ^c^Cirrhosis diagnosed by liver biopsy or transient elastography. Interpretative TE cut-offs from the Gambia and Senegal were applied in this study for cirrhosis (F4) >9.4 kPa and significant fibrosis (F2) >7.9 kPa. ^d^If HBV DNA quantification is not available, treatment may be considered based on persistently abnormal ALT alone

**Supplementary Appendix 3:** **Validation of a quantitative in-house hepatitis B DNA PCR**

*Sample and standards*

The assay used plasma, collected in K2 EDTA tubes, separated by centrifugation at 1500xg for 10 minutes prior to storage at -80°C. Plasma samples were restored to room temperature prior to testing. The reference standard for absolute quantification was the 4^th^ World Health Organization (WHO) International Standard for HBV DNA for Nucleic Acid Amplification Techniques (National Institute for Biological Standards and Control, Potters Bar, United Kingdom). A patient sample positive for HBsAg and HBeAg by ELISA (Monolisa HBsAg ULTRA and Monlisa HBe Ag-Ab, Bio-Rad, Marnes-la-Coquette, France) was aliquoted and used as a standard, and was quantified and calibrated using the WHO standard.

*Nucleic acid extraction*

Nucleic acid was extracted using the Qiamp DNA Mini kit (Qiagen, Hilden, Germany), according to the manufacturer’s instructions for viral DNA extraction using 200 µl of plasma. Extracted nucleic acid was eluted in 60µl of Tris-EDTA buffer, pH 8.0 (Fisher Scientific, Loughborough, United Kingdom) with added poly-A carrier RNA at 10ng/µl (Qiagen). Following extraction, eluted DNA was stored on ice while awaiting PCR. A negative control consisting of Tris-EDTA buffer from the extraction bench was added to every reaction plate.

*Primer and probes*

The primers and probe are from previously published assays targeting a conserved region of the hepatitis B surface gene (Table S1).^1,2^

**Table S1: Details of primers and probe**

| **Primer/probe** | **Sequence 5’ - 3’** |
| --- | --- |
| HBV_Forward | GTG TCT GCG GCG TTT TAT CA |
| HBV_Reverse | GAC AAA CGG GCA ACA TAC CTT |
| HBV_Probe | 5’ FAM- CC TCT KCA T/ZEN/C CTG CTG CTA TGC CTC ATC – 3’ IBFQ’ |

*Preparation of Assay*

PCR plates were prepared using a pipetting robot (Qiagility, Qiagen). Reactions included 25μl TaqMan universal mastermix (ThermoFisher, USA), 400nM of forward and reverse primers and 200nM of the probe, with 15μl of extracted DNA template, with molecular biology grade water added to a total reaction volume of 50μl. The QuantStudio 7 Flex Real-Time PCR System (Applied Biosystems, Waltham, MA, USA) was used with a 200µl well qPCR plate with the following conditions: 10 minutes at 95°C, followed by 42 cycles of 15 seconds at 95°C and 1 minute at 60°C. ROX passive reference dye was used.

*Quantification of Standard*

The WHO standard was reconstituted using molecular biology grade water and diluted 1:10 from to 955,000 to 95.5 IU/ml. The patient sample was diluted in six serial 1: 10 dilutions and run alongside the WHO standard. Dilutions were run in triplicate. (Figure S1)

The patient sample diluted x10^-4^, was within the measurable linear range of the WHO standard dilutions and was quantified as mean 4.57 log_10_ IU/ml, and 4.58 log_10_ IU/ml from six reactions from two separate experiments, with a mean concentration of 4.58 log_10_ IU/ml. The patient sample was assigned a value of 8.58 log10 IU/ml. Serial dilutions of the reference sample were measured in duplicate on each PCR plate.

*Linearity*

The linear range of the assay was assessed with triplicate serial 1:10 dilutions of sample P004 on three separate experiments from 8.58 log_10_ IU/ml to 0.58 log_10_ IU/ml. The assay demonstrated linearity across this range (Figure S2). Efficiency was 93.1% and R^2^ was 0.996

*Lower limit of detection and quantification*

Repeated serial dilutions of the patient sample were analysed to determine the lower limit of quantification (LLQ) and detection (LLD) of the assay. A probit analysis demonstrated a LLQ of 34 IU/ml (95% confidence interval 23- 64) and LLD of 30 IU/ml (95% CI 20- 63) (Table S2)

**Table S2: Analysis of lower limit of quantification and detection of assay using serial dilutions of sample P004**

| Quantity (log_10_ IU/ml) | Quantity (IU/ml) | Detected | Linear quantification | Number of replicates |
| --- | --- | --- | --- | --- |
| 3.58 | 3784 | 30 | 30 | 30 |
| 2.58 | 378 | 31 | 31 | 31 |
| 2.28 | 189 | 25 | 25 | 25 |
| 1.98 | 95 | 30 | 30 | 30 |
| 1.58 | 38 | 36 | 35 | 36 |
| 1.28 | 19 | 29 | 25 | 30 |
| 0.98 | 9 | 19 | 19 | 30 |
| 0.68 | 5 | 13 | 11 | 30 |

**References:**

1. Garson JA, Grant PR, Ayliffe U, Ferns RB, Tedder RS. Real-time PCR quantitation of hepatitis B virus DNA using automated sample preparation and murine cytomegalovirus internal control. Journal of virological methods 2005; 126(1-2): 207-13.

2. Ghosh S, Sow A, Guillot C, et al. Implementation of an in-house quantitative real-time polymerase chain reaction method for Hepatitis B virus quantification in West African countries. Journal of viral hepatitis 2016.

**Figure S1: Serial dilutions of the 4^th^ WHO International standard and reference patient sample**


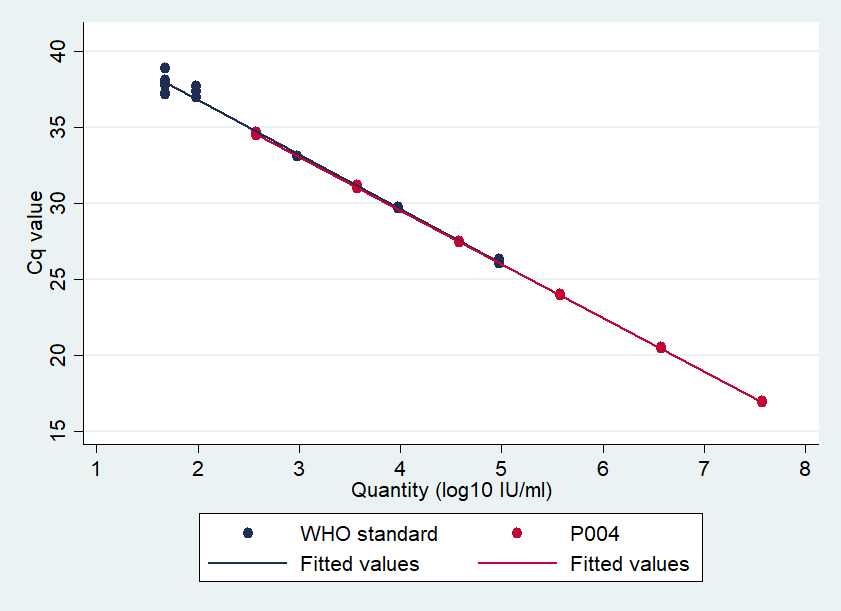


**Figure S2: Assessment of Linearity of HBV DNA assay with serial 1:10 dilution of the patient reference sample**


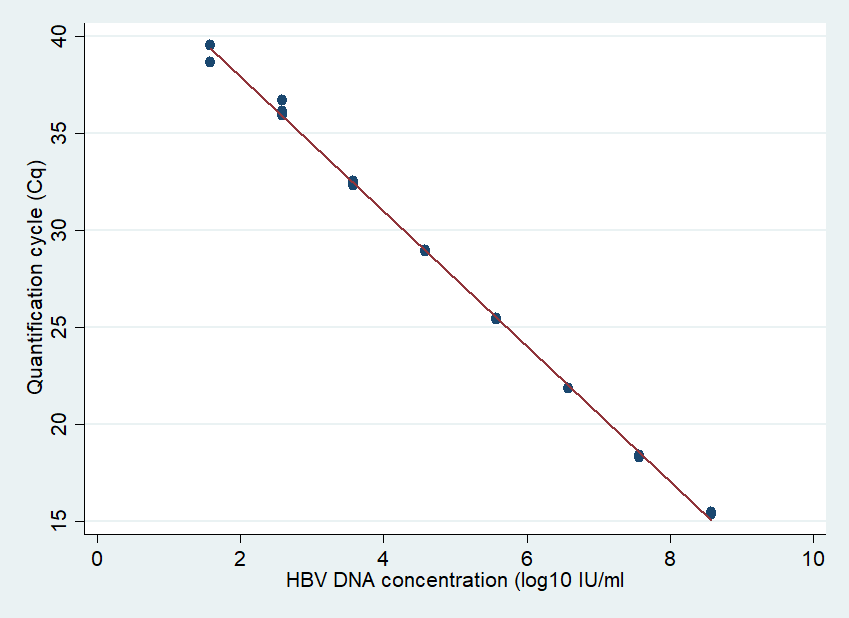


**Supplementary Appendix 4:** **Socioeconomic status questionnaire**

| **Question** | **Response options recorded** |
| --- | --- |
| Number of rooms in house | Number |
| What kind of toilet facility is currently in use within the household? | Household use only  Shared with neighbours  Public  They don’t have one |
| Type of toilet used | Flush/pour flush  Pit latrine with slab  Pit latrine with wood/soil floor  Open defaecation  Unwilling to answer |
| Primary source of drinking water | Piped to house  Public tap outside house  Private tap outside house  Public tap/standpipe  Borehole/Well (protected)  Unprotected |
| Does the household keep animals? | Yes/ No |
| Does the household keep companion animal(s)? | Yes/ No |
| Does the household keep livestock animal(s)? | Yes/ No |
| Does the household ever use firewood for fuel? | Yes/ No |
| Concerning your household food consumption over the past  one month, which one of the following is true? | Less than adequate for Household  needs  Just adequate for Household needs  More than adequate for Household  needs |
| What does the head of the household sleep on? | Bed and Matress  Bed and Matt (grass)  Bed alone  Mattress on floor  Matt (grass) on floor  Cloth/Sack on floor  Floor (nothing else)  Other |
| Does your household own a radio ? | Yes/ No |
| Does your household own an iron? | Yes/ No |
| What type of construction materials are used for the house? | Permanent  Semi-permanent  Traditional |
| The floor of the main house is predominantly made of what  material? | Sand  Smoothed Mud  Smoothed Cement  Wood  Tile  Other |

**Supplementary Appendix 5A**: **Satellite image of census data, area boundaries and study locations in Ndirande township^a^ 5B: Map of serological survey indicating GPS location of participants^b^**

**
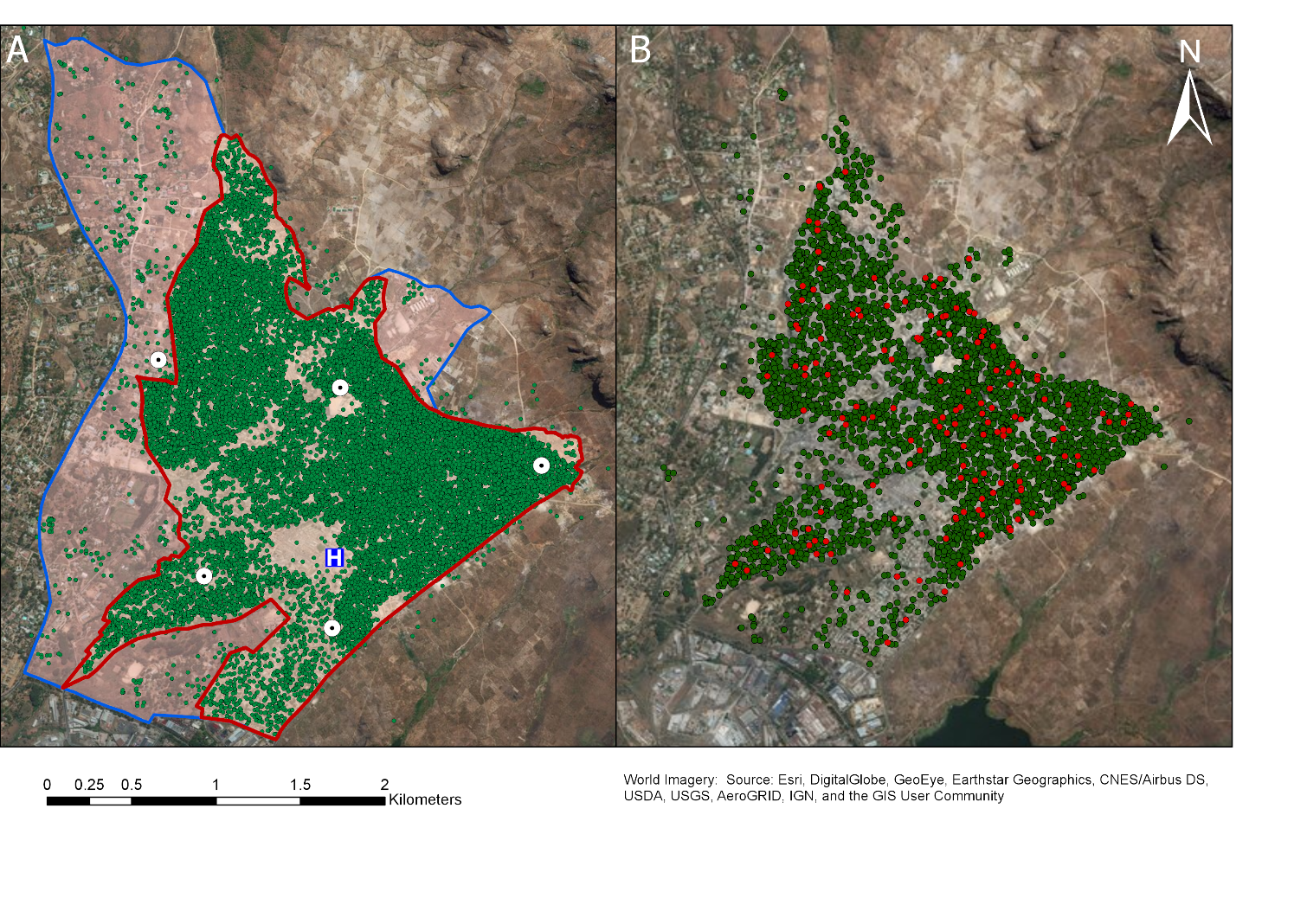
**

^a^5A:Green points show GPS locations of 97386 individuals recorded on the demographic census. Blue boundaries show the 7.5km^2^ outer health surveillance area and red boundaries define the 4.5km^2^ central region. “H” symbol denotes the health centre. White circle markers show locations where the community evaluation study was conducted. ^b^5B:Points show locations of 6073 participants in the serosurvey. Red markers indicate participants who tested positive for HBsAg by ELISA. All points indicating HBsAg-positive individuals (panel 5B) are randomly scattered for confidentiality. No spatial clustering of HBsAg-positive individuals was observed by Getis-Ord Gi* and Anselin Local Moran’s I statistics.

**Supplementary Appendix 6: Prevalence of hepatitis B surface antigen (HBsAg) among serosurvey participants**

| **Age (years)** | **Male** | | **Female** | | **Total** | |
| --- | --- | --- | --- | --- | --- | --- |
|  | **Frequency** | **% (95% CI)** | **Frequency** | **% (95% CI)** | **Frequency** | **% (95% CI)** |
| 0–4 | 2/270 | 0.7 (0.2 – 2.7) | 0/239 | 0.0 (0.0 – 1.6) | 2/509 | 0.4 (0.1 – 1.4) |
| 5–9 | 2/695 | 0.3 (0.0 – 1.0) | 2/670 | 0.3 (0.1 – 1.1) | 5/1365 | 0.4 (0.2 – 0.9) |
| 10–14 | 1/392 | 0.3 (0.0 – 1.4) | 2/434 | 0.5 (0.1 – 1.7) | 3/826 | 0.4 (0.1 – 1.1) |
| 15–29 | 20/496 | 4.0 (2.6 – 6.1) | 24/734 | 3.3 (2.2 – 4.8) | 44/1230 | 3.6 (2.7 – 4.8) |
| 30–39 | 25/281 | 8.9 (6.0 – 12.8) | 38/630 | 6.0 (4.4 – 8.2) | 63/911 | 6.9 (5.4 – 8.8) |
| 40–49 | 10/187 | 5.3 (2.9 – 9.6) | 14/314 | 4.5 (2.7 – 7.3) | 24/501 | 4.8 (3.2 – 7.0) |
| 50–59 | 5/147 | 3.4 (1.5 – 7.7) | 6/242 | 2.5 (1.1 – 5.3) | 11/389 | 2.8 (1.6 – 5.0) |
| 60–69 | 5/84 | 6.0 (2.6 – 13.2) | 3/146 | 2.1 (0.7 – 5.9) | 8/230 | 3.5 (1.8 – 6.7) |
| ≥70 | 1/66 | 1.5 (0.3 – 8.1) | 0/46 | 0.0 (0.0 – 7.7) | 1/112 | 0.8 (0.2 – 4.9 |
| All | 71/2618 | 2.7 (2.2 – 3.4) | 89/3455 | 2.6 (2.1 – 3.2) | 161/6073 | 2.7 (2.3 – 3.1) |

**Supplementary Appendix 7:** Sensitivity analyses for estimates of vaccine impact

| **Analysis** | **Risk ratio, odds ratio or incidence risk ratio**  **(95% CI)** | **P value** | **Vaccine impact estimate**  **(% (95% CI))** |
| --- | --- | --- | --- |
|  |  |  |  |
| Model 1: Binomial log linear regression (risk ratio) | 0.04 (0.06- 0.29) | 0.001 | 95.9 (70.7 – 99.4) |
| Sensitivity analysis 1: Binomial logistic regression (odds ratio) | 0.04 (0.06 – 0.29) | 0.001 | 96.0 (71.3 – 99.4) |
| Sensitivity analysis 2: Poisson regression analysis (Incidence risk ratio) | 0.06 (0.01 – 0.41) | 0.004 | 94.2 (59.2 – 99.2) |
| Sensitivity analysis 3: Model 1, adjusted for age^b^ | 0.03 (0.001 – 0.41) | 0.009 | 97.1 (58.5 – 99.8) |
| Sensitivity analysis 4: Model 1, adjusted for age and birth cohort (2-year intervals)^b,c,d^ | 0.04 (0.002 – 0.52) | 0.015 | 96.5 (48.2 – 99.8) |

^a^Comparison of HBsAg prevalence among individuals born 5 years before and after vaccine introduction (incidence risk ratio), by date of birth. ^b^Adjustment for participant age at time of sampling as a continuous variable, in addition to birth date. ^c^Additional adjustment for birth cohort in 2-year intervals. ^d^ Wald likelihood test for comparison between sensitivity analysis 3 and 4=

**Supplementary Appendix 8:** Impact of post-stratification iterative fitting weights for geographic region on prevalence and vaccine impact estimates^a^

| **Population** | **Population standardised prevalence adjusted for age and sex** | **Population standardised prevalence adjusted for age, sex and geographic area** |
| --- | --- | --- |
|  | % (95% CI) | % (95% CI) |
| All individuals | 3.2 (2.7 – 3.7) | 3.2 (2.7, 3.8) |
| **Birth date relative to vaccine introduction**^b^ |  |  |
| 5 yr before vaccine | 2.9 (1.8 – 4.7) | 2.9 (1.8 – 4.8) |
| 5 yr after vaccine | 0.1 (0.02 – 0.8) | 0.1 (0.02-1.0) |
| 10 yr before vaccine | 3.6 (2.5 – 5.1) | 3.5 (2.4 – 5.0) |
| 10 yr after vaccine | 0.2 (0.1 – 0.6) | 0.2 (0.1 – 0.6) |
| All before vaccine | 5.1 (4.3 – 6.0) | 5.1 (4.3 – 6.0) |
| All after vaccine | 0.3 (0.2 – 0.6) | 0.3 (0.2 – 0.6) |
| **Vaccination status**  **for age ≤10 years**  Completed 3 doses | 0.2 (0.1 – 0.8) | 0.2 (0.1 -0.7) |
| Unknown status ^c^ | 0.6 (0.2 – 1.4) | 0.6 (0.2 – 1.4) |
| Incomplete ^d^ | - | - |

^a^To assess for the potential impact of variation in geographic response rates between geographic areas in the serosurvey this sensitivity analysis examines the effect of including post-stratification iterative fitting for geographic area (based on 12 health surveillance areas in the township) to adjust prevalence for geographic variation in response rates, in addition to adjustment for age and sex.

**Supplementary Appendix 9:** Comparison of characteristics of participants included in clinical evaluation of HBV treatment eligibility with potentially eligible non-participants

| **Characteristics** | **Included**  **n= 94** | **Non-participants**  **N=56** | **P value** |
| --- | --- | --- | --- |
| Age (years), median (IQR) | 35 (29, 41) | 34 (28, 42) | 0.78 |
| Sex (female), n (%) | 49 (52) | 35 (63) | 0.22 |
| Marital status^a^, n(%) |  |  | 0.09 |
| Single | 21 (23) | 7 (14) |  |
| Married | 64 (69) | 34 (68) |  |
| Divorced/separated | 3 (3) | 7 (14) |  |
| Widowed | 5 (5) | 2 (4) |  |
| Education, n(%) |  |  | 0.01 |
| None | 2 (2) | 1 (2) |  |
| Primary | 26 (28) | 25 (47) |  |
| Secondary | 60 (64) | 22 (42) |  |
| Vocational | 1 (1) | 4 (8) |  |
| University | 5 (5) | 1 (2) |  |
| Employment, n(%) |  |  | 0.03 |
| Unemployed | 17 (18) | 17 (32) |  |
| Student | 10 (11) | 1 (2) |  |
| Self-employed | 47 (50) | 19 (36) |  |
| Paid employee | 35 (24) | 16 (30) |  |
| Retired | 1 (1) | 0 (0) |  |
| Socioeconomic status^c^ |  |  | 0.02 |
| Highest quintile | 10 (11) | 5 (9) |  |
| 2^nd^ highest quintile | 25 (27) | 6 (11) |  |
| Middle quintile | 22 (23) | 12 (21) |  |
| 2^nd^ lower quintile | 12 (13) | 19 (34) |  |
| Lowest quintile | 25 (27) | 14 (25) |  |

^a^See Figure 1 for a list of reasons for non-participation. ^b^Marital status was available for 50/56, and education and employment status for 53/56, of those excluded.

**Supplementary Appendix 10:** Parametric bootstrapping procedure to estimate confidence intervals for national estimates of treatment eligibility^a^

| **Metric** | **Prevalence**  **(95% CI)** | | **Number**  **95% CI** | |
| --- | --- | --- | --- | --- |
| HIV prevalence in national population aged 15-64 | 10.6% | (9.9 - 11.2) | 1,043,587 | (974,671 – 1,102,658) |
| Estimated HIV negative population aged ≥15 years | 89.4% | (88.8 – 90.1) | 8,801,575 | (8,742,504 – 8,870,491) |
| HBV prevalence among general population aged ≥15 years (number presented among HIV-negative population) | 5.1% | (4.3 – 6.1) | 448,880 | (378,392-537,340) |
| Eligible for treatment according to EASL 2017 criteria | 5.7% | (1.6-14.2) | 25,586 | (7,172 – 65,519) |
| Eligible for treatment according to AASLD 2018 criteria | 8.7% | (3.3-18.0) | 39,053 | (14,737 – 83,426) |
| Eligible for treatment according to WHO 2015 criteria | 2.9% | (0.7-10.0) | 13,018 | (3,142- 46,030) |

^a^The population aged ≥15 years was 9,845,162 in the 2018 national census. Estimates for people with HBV eligible for HBV treatment in the national population were computed by multiplying the census population over 15 years by (1- national HIV prevalence estimates for the population aged 15-64) by the proportion of the HBsAg-positive, HIV negative clinical evaluation population eligible for treatment in this study according to EASL, AASLD and WHO criteria respectively. We derived 95% confidence intervals by finding the beta distributions for the 2.5^th^ and 97.5^th^ percentiles matching the 95% confidence intervals for HIV negative prevalence, HBV positive prevalence and proportion eligible for HBV treatment. We generated N=10,000,000 prevalence samples using parametric bootstrapping for each distribution and multiplied these. We used the empirical 2.5^th^ and 97.5^th^ quantiles of this sample as the upper and lower bounds for the 95% CI, and used the product to derive the central estimate. Analyses were conducted in R version 4.0.2. (R Foundation for Statistical Computing) using the bootComb package: Henrion, M. (2020), bootComb: Combine Parameter Estimates via Parametric Bootstrap, R package version 1.0.0, <https://cran.r-project.org/package=bootComb>.
